# Ice nucleation activity in various tissues of *Rhododendron* flower buds: their relevance to extraorgan freezing

**DOI:** 10.3389/fpls.2015.00149

**Published:** 2015-03-25

**Authors:** Masaya Ishikawa, Mikiko Ishikawa, Takayuki Toyomasu, Takayuki Aoki, William S. Price

**Affiliations:** ^1^Division of Plant Sciences, National Institute of Agrobiological SciencesTsukuba, Japan; ^2^Genetic Resources Center, National Institute of Agrobiological SciencesTsukuba, Japan; ^3^Nanoscale Organization and Dynamics Group, University of Western Sydney, PenrithNSW, Australia

**Keywords:** anti-nucleation activity, azalea, osmolality, cold hardiness, extraorgan freezing, flower buds, ice nucleation activity, *Rhododendron*

## Abstract

Wintering flower buds of cold hardy *Rhododendron japonicum* cooled slowly to subfreezing temperatures are known to undergo extraorgan freezing, whose mechanisms remain obscure. We revisited this material to demonstrate why bud scales freeze first in spite of their lower water content, why florets remain deeply supercooled and how seasonal adaptive responses occur in regard to extraorgan freezing in flower buds. We determined ice nucleation activity (INA) of various flower bud tissues using a test tube-based assay. Irrespective of collection sites, outer and inner bud scales that function as ice sinks in extraorgan freezing had high INA levels whilst florets that remain supercooled and act as a water source lacked INA. The INA level of bud scales was not high in late August when flower bud formation was ending, but increased to reach the highest level in late October just before the first autumnal freeze. The results support the following hypothesis: the high INA in bud scales functions as the subfreezing sensor, ensuring the primary freezing in bud scales at warmer subzero temperatures, which likely allows the migration of floret water to the bud scales and accumulation of icicles within the bud scales. The low INA in the florets helps them remain unfrozen by deep supercooling. The INA in the bud scales was resistant to grinding and autoclaving at 121^∘^C for 15 min, implying the intrinsic nature of the INA rather than of microbial origin, whilst the INA in stem bark was autoclaving-labile. Anti-nucleation activity (ANA) was implicated in the leachate of autoclaved bud scales, which suppresses the INA at millimolar levels of concentration and likely differs from the colligative effects of the solutes. The tissue INA levels likely contribute to the establishment of freezing behaviors by ensuring the order of freezing in the tissues: from the primary freeze to the last tissue remaining unfrozen.

## Introduction

Wintering cold hardy woody plant tissues display diverse freezing behaviors under subfreezing temperatures, such as extracellular freezing (e.g., bark), deep supercooling (e.g., xylem ray parenchyma) and extra-organ freezing (e.g., flower buds and leaf buds) (Ishikawa and Sakai, 1982; Sakai and Larcher, 1987). These types of freezing behavior (freezing strategies) are specific to species and tissues and are considered to play key roles in their cold hardiness mechanisms. However, the underlying mechanisms that determine the tissue- and species-specific freezing behaviors are poorly understood.

In the extraorgan freezing of woody plant flower buds, flower primordia (florets) remain stably unfrozen whereas bud scales freeze first, working as an ice sink to withdraw water from the florets to the scales when cooled at naturally occurring cooling rates (Ishikawa and Sakai, 1982). This enhances the deep supercooling capability of the florets (consequently further avoiding lethal breakdown of floret supercooling) with concomitant ice accumulation in the scale tissues. The extent of water migration (from florets to scales) and the resultant supercooling enhancement differ considerably depending upon species and the size of supercooled organs, resulting in further classification of extraorgan freezing into Type I ∼ III (Ishikawa and Sakai, 1982). The most important difference between extraorgan freezing and extracellular freezing is that the ice barrier (the boundary between the supercooled and frozen parts) is located at the tissue/organ level in the former whereas at the cellular level (cell walls and/or plasma membranes working as the ice barrier) in the latter. This results in the differences in the rate of cellular dehydration (extracellular freezing >> extraorgan freezing) and in the distribution of icicles within an organ.

A number of questions have been raised regarding the mechanism of extraorgan freezing in flower buds (Ishikawa and Sakai, 1985): (1) what makes florets remain stably and deeply supercooled, (2) through where does the floret water migrate to bud scales, (3) where and how ice propagation into unfrozen florets is prevented, (4) why bud scales freeze first in spite of their lower water content, (5) whether floret cells have the ability to undergo extracellular freezing. Question (2) has been answered by the insertion of silicone oil into the space around the floret (Ishikawa and Sakai, 1985) and by the use of NMR micro-imaging (Ishikawa et al., 1997; Price et al., 1997): the floret water migrates through the pedicel (the basal part of the floret). Question (3) has been frequently addressed and yet localization and the identity of the ice barrier (biochemical, structural in nature or just formation of a dehydrated zone) remain to be elucidated (Quamme, 1978; Ishikawa and Sakai, 1981, 1985; Ashworth, 1992; Chalker-Scott, 1992; Kader and Proebsting, 1992; Wisniewski et al., 2009; Kuprian et al., 2014). But, Questions (1), (4), and (5) have not been seriously investigated to our knowledge.

Amongst the important unsolved questions about extraorgan freezing is why bud scale tissues freeze before the florets despite the floret tissues having much higher water content (e.g., 200 ∼ 250% dry weight) than the bud scales (e.g., 100 ∼ 150% dry weight) and why the florets remain unfrozen through stable deep supercooling (Ishikawa and Sakai, 1981, 1985). The initiation of freezing in the bud scales prior to florets seems crucial to properly accommodate ice in the ice sink (bud scales) and to create a motive force that allows the floret water to be withdrawn slowly to the bud scales, that in turn enhances the supercooling capability of the florets (Ishikawa and Sakai, 1985). Ishikawa and Sakai (1985) postulated two factors that contribute to the initiation of freezing in the bud scales: (1) a temperature gradient (lower in the bud scales than in the florets) formed during the cooling process since the scales are located at the outer surface; (2) the presence of high ice nucleation activity (INA) in the bud scales, either of bacterial or intrinsic origin and the lack of such activity in the florets. One of the objectives of the present paper is to substantiate postulate (2) by determining the INA levels of flower bud parts of *Rhododendron japonicum*. The INA of a tissue can be defined as the capability of that tissue to cause heterogeneous ice nucleation in a controlled environment and can be determined using a test tube-based assay (Kishimoto et al., 2014a,b). We also measured the osmolality of flower bud tissues as another possible factor involved in the supercooling capability and ice nucleation of the tissues. Flower buds of *R. japonicum* were selected (revisited) as the plant material as they display most pronounced floret water migration to the scales among *Rhododendron* flower buds, undergoing typical Type II extraorgan freezing (Ishikawa and Sakai, 1981) and are considered as a good model system for studying the mechanism of extraorgan freezing.

Another objective of the study is to characterize the INA in this plant as there is only limited knowledge on this trait (INA of plant origins) including whether it is of plant or microbial origins. To downplay the effect of collection sites, we first checked if flower buds collected in different sites undergo extraorgan freezing in similar manners and show similar distribution of INA in the tissues. Next, we determined the effects of tissue amount, grinding into powder and autoclaving (121°C for 15 min) on the levels of INA in the bud scales. During this process, we found extremely heat stable INA (likely of plant intrinsic origin) in bud scales and the nearly universal occurrence of anti-nucleation activity (ANA), which is defined as the ability to suppress the INA, in the supernatant of autoclaved tissues of different kinds. Thirdly, we followed seasonal changes in the INA (plus their heat stability) and osmolality of the flower bud tissues. For comparison purposes, localization and characteristics of INA in stem tissues were also determined. These processes also allowed our test tube INA assay (Kishimoto et al., 2014a,b) to be evaluated for their effectiveness and applicability. Lastly, we attempted to detect the occurrence of microorganisms associated with bud scales. These results are discussed in regard to the intrinsic freezing behavior of the flower bud tissues including the mechanism of extraorgan freezing, the origin (microbial or intrinsic) of INA and the occurrence of two distinct types (plus their development) of INA in flower bud tissues.

## Materials and Methods

### Plant Materials

Stems with terminal flower buds were collected from August until May, randomly from 1.5 ∼ 3 m tall *R. japonicum* plants in natural populations (composed of approximately 2000 plants growing in a sunny mountain area of 1100 m elevation) in Tochigi Prefecture (N37.12°, E140.18°), approximately 150 km north of Tokyo, Japan, unless otherwise specified. This species is commercially available for gardening and cultivated in cool temperate zones in Japan and worldwide. To check the effect of collection sites or cultivation sites, they were also sampled from plants cultivated for more than 10 years in three other sites: Botanical Garden of National Science Museum, Tsukuba (N36.08°, E140.08°), approximately 50 km northeast of Tokyo; Botanical Garden of Hokkaido University, Sapporo, Hokkaido (N43.06°, E141.35°); Horticulture Center of Asahikawa City, Hokkaido (N43.77°, E142.37°).

### Differential Thermal Analysis (DTA)

Excised flower buds on stems with a length of about 1 cm were cooled at 3°C/h from 5°C down to -40°C in a programmable deep freezer (Model FPR-120S Fuji Ika Sangyo Co. Ltd.) unless otherwise specified. Exothermic events were detected with a copper-constantan thermocouple attached to the surface of the outer bud scales and amplified 40–100 times with an amplifier (Micro Volt Meter AM-1001, Ohkura Electric Co., Japan) prior to recording (Ishikawa and Sakai, 1981). Six or more samples were used for each determination, but only a typical profile is shown.

### Observation of Icicle Localization Under A Dissecting Microscope

Several *R. japonicum* shoots (approximately 20 cm-long) with terminal flower buds collected in early December were enclosed in polyethylene bags with a small amount of snow. The bags were placed in an insulated cool box and cooled at 5°C/day to -15°C in a programmable freezer, then transferred to a cold room (-15°C) without thawing the samples. They were stored there for 3 days before the localization of icicles was determined by visually observing the dissected flower bud tissues under a binocular microscope at -15°C.

### Determination of Ice Nucleation Activity (INA)

The INA of flower bud tissues was measured with a test tube assay (Kishimoto et al., 2014a,b). Briefly, flower buds of *R. japonicum* were carefully dissected into various parts with sterilized forceps or knives: outer scales, inner scales and florets. Current year stem pieces (7 mm-long) and their comprising tissues (bark, xylem, and pith) were prepared in a similar manner. Each tissue part was put into 2 ml sterilized Milli-Q water in TPX test tubes (40–50 replicates for a sample) with a lid of transparent polyester film (all the equipment and water were sterilized by autoclaving at 121°C for 15 min before use). All these processes were done in a laminar flow chamber to avoid contamination from airborne ice nuclei of microbial or other origin. The tubes were cooled in a stepwise manner in a precision-controlled bath from 0 to -20°C in 1°C decrements. Each 1°C step took approximately 5 min. The tubes were maintained at each temperature for 20 min before counting the number of frozen tubes. Frozen or unfrozen tubes were easily recognized by viewing the tube content through the transparent lid using backlighting introduced to the bath via an optical fiber. This allowed the tubes to remain untouched until freezing and reduced unnecessary vibration of the tubes, which otherwise may induce erroneous freezing of the tubes. We could obtain highly reproducible results using this method (Ishikawa, 2014; Kishimoto et al., 2014a,b). In some experiments, the amount of tissue placed in a tube was increased to check the quantitative effect of sample amount on the levels of INA.

Since ice nucleation events involve probability, the number of tubes used in an INA assay is important to obtain valid data. To make a 1°C INA difference statistically significant, at least 40–50 tubes are required. The results are reported as the cumulative number of frozen tubes vs. temperature (e.g., **Figure 4**). To represent the INA of a sample, the median ice nucleation temperature (INT), at which 50% of the tubes froze is used rather than the mean INT, since the latter is prone to be affected by any erroneously high or low INT value. To indicate the variance of INT, ±SD is tabulated in **Tables 1** and **2**, which statistically means that two-thirds of the INT values are within 1 × SD range. If the sample contains numerous good ice nucleators, the median INT is shifted to warm temperatures with smaller SD. If the sample contains low amount of ice nucleators, the median INT is shifted to lower temperatures with larger SD. This relationship can be estimated from the INA determination of serially diluted ice nucleating bacteria solutions using the same 2 mL test tube assay system (data not shown). Occasionally, one might get the INT values at warm temperatures with large SD. This likely arises from the uneven occurrence of ice nucleators in the samples or from the large variance in the sample mass in the tubes.

**Table 1:** Ice nucleation activity (INA) in various tissues of *Rhododendron japonicum* flower buds collected from various sites.

Collection sites and dates	Ice nucleation activity (median INT), ^∘^C		
Tissues	Intact	AC	AC/WC
***R. japonicum* Asahikawa (October 24)**			
OS	-5.2 ± 0.3	-6.8 ± 0.5	-5.5 ± 0.4
IS	-6.7 ± 1.4	-8.8 ± 1.4	-7.8 ± 1.1
FL	-16.8 ± 1.7	-16.0 ± 1.4	-16.8 ± 1.3
***R. japonicum* Sapporo BG (December 15)**			
OS	-6.0 ± 0.5	-6.6 ± 0.3	-4.9 ± 0.8
IS	-7.2 ± 0.8	-8.2 ± 1.4	-8.2 ± 1.5
FL	-16.3 ± 1.9	-17.3 ± 1.6	-17.3 ± 2.0
***R. japonicum* Tsukuba (November 22)**			
OS	-5.6 ± 0.4		-5.9 ± 0.7
IS	-6.6 ± 1.0		-7.8 ± 1.2
FL	-14.8 ± 1.4		-17.2 ± 1.5
***R. japonicum* Tochigi (October 24)**			
OS	-5.2 ± 0.4	-5.9 ± 0.5	-5.4 ± 0.4
IS	-6.7 ± 0.7	-8.8 ± 1.3	-7.8 ± 1.3
FL	-15.0 ± 1.8	-17.7 ± 1.8	-17.3 ± 1.7
***R. japonicum* Tochigi (December 2)**			
OS	-5.7 ± 0.3		-5.5 ± 0.4
IS	-6.4 ± 0.7		-6.6 ± 1.0
FL	-15.0 ± 1.5		-17.3 ± 1.7
Stem	-6.1 ± 0.8		-12.0 ± 0.8
Bark	-6.2 ± 1.1		-13.3 ± 1.0
Xylem	-12.3 ± 1.3		-13.8 ± 0.9
Pith	-12.4 ± 1.2		-12.5 ± 0.8

INA was determined using the revised test tube assay and expressed as the 50% ice nucleation temperature (median INT). Intact, tubes with 2 mL of autoclaved Milli-Q water plus intact specimens were used for the INA assay. After the assay, the tubes with samples were autoclaved at 121^∘^C for 15 min and directly used for a further INA assay (AC) or after replacing the supernatant with fresh cold sterilized water and used for the INA assay (AC/WC). A single outer scale (OS), inner scale (IS) or floret (FL) was added to each test tube for INA assay. The data are presented in the form of median INT ± SD, so that the variance of the INT can be seen (two-thirds of the INT values are within the SD range).

**Table 2:** Effect of sample amount, grinding and autoclaving on the ice nucleation activity in bud scales of wintering *R. japonicum* flower buds.

*R. japonicum* Tochigi (November 22)	Ice nucleation activity (median INT), ^∘^C		
	Intact	AC	AC/WC
IS × 1	-6.6 ± 1.0	-7.8 ± 1.2	
IS × 4	-5.9 ± 0.6	-6.6 ± 0.7	
OS × 1	-5.6 ± 0.4	-7.7 ± 0.5	
OS × 4	-5.2 ± 0.4	-6.6 ± 0.5	
OS powder × 1	-5.5 ± 0.4	-7.4 ± 0.2	-5.8 ± 0.4
OS powder × 4	-5.3 ± 0.5	-6.9 ± 0.5	-5.4 ± 0.3
OS powder × 16			-5.3 ± 0.2
OS powder × 32			-4.7 ± 0.2

Intact, tubes with 2 mL of autoclaved Milli-Q water plus intact specimens were used for INA assay. After the assay, the tubes with samples were autoclaved at 121^∘^C for 15 min and directly used for another INA determination (AC) or after replacing the supernatant with fresh cold sterilized water and used for INA assay (AC/WC). A single or four pieces of outer scale (OS), inner scale (IS) or the equivalent amount of ground tissue powder was added to each test tube for the INA assay unless otherwise specified. The data are presented in the form of median INT ± SD, so that the variance of INT can be seen (two-thirds of the INT values are within the SD range).

### Effect of Grinding and Autoclaving on the INA of Flower Bud Scales

To check heat stability of the INA in the samples, the tubes containing flower bud parts after being used for INA determination were autoclaved for 15 min at 121°C. After cooling, the autoclaved tubes with samples were utilized for a second INA determination. Following thawing, the samples were placed in a laminar flow chamber and the leachate was carefully discarded with Pasteur pipettes without removing the specimen. Then, a 2 mL aliquot of autoclaved Milli-Q water was added to each tube aseptically prior to another INA determination (i.e., the third determination). In some cases, the second INA determination was omitted and the leachate in the autoclaved tubes containing samples was directly replaced with fresh sterilized water and then the INA determined.

Outer scales excised from forty flower buds of *R. japonicum* collected in late November were frozen using liquid nitrogen and ground with a mortar and pestle into powder. The amount equivalent to one or four outer scales was put into test tubes and used for the INA assay. Following the assay, the tubes with powder were autoclaved at 121°C for 15 min. After cooling, the tubes were used for a further INA assay. Following the assay, the supernatant was aseptically discarded and replaced twice with 2 mL fresh autoclaved Milli-Q water before the third INA assay.

### Determination of Water Content

The dry weight of flower bud parts was determined by oven-drying at 70°C for 2 days and the water content was expressed as the percentage of dry weight unless otherwise noted. The water content of flower bud scales and florets during slow cooling (5°C/day) to subfreezing temperatures was determined by dissecting the flower bud parts in cold rooms (-10 or -15°C) without thawing as detailed previously (Ishikawa and Sakai, 1981).

### Determination of Osmolality of Various Flower Bud Parts

Each part excised from 20 or more *R. japonicum* flower buds was placed in disposable tubes and subjected to repeated freeze-thaw cycles in liquid nitrogen and 25°C water. Then, the tissues were transferred into a disposable syringe plugged with glass wool. The osmolality of the cell sap squeezed from the syringe was determined with a vapor pressure osmometer (Wescor Inc., Logan, UT, USA).

### Detection of Microorganisms (Mainly Fungi) Associated with Wintering Flower Bud Scales of *R. japonicum*

To remove microbial flora loosely associated with the surface of the plant specimens, ten whole flower buds with 5 mm of stem were vigorously washed in 10 mL of sterilized 0.005% detergent (di-*iso*-octyl sodium sulfosuccinate) solution by vortexing for 1 min. The detergent solution was replaced with fresh solution. This washing process was repeated five times, followed by rinsing three times with 10 mL of sterilized water by vortexing for 1 min each time. This washing technique (Aoki et al., 1992) is a modification of the method developed by Harley and Waid (1955). Then the flower buds were blotted aseptically on filter paper in a sterilized petri dish to remove surface water and left there overnight. Subsequently, the outer and inner bud scales were carefully excised using clean forceps in a laminar flow chamber and each single scale was placed on half-strength cornmeal agar medium (Difco Laboratories, Detroit, MC, USA) prepared in a petri dish. The petri dish were incubated for 14 days at 25°C before observing colony development of fungi and microorganisms around the specimen and scoring them (+ or -). This method detects microorganisms rather tightly associated with plant tissues including endophytes, if any, which can grow on this medium (Aoki et al., 1992).

## Results and Discussion

### Freezing Behavior of *R. japonicum* Flower Buds Revisited

**FIGURE 1:**
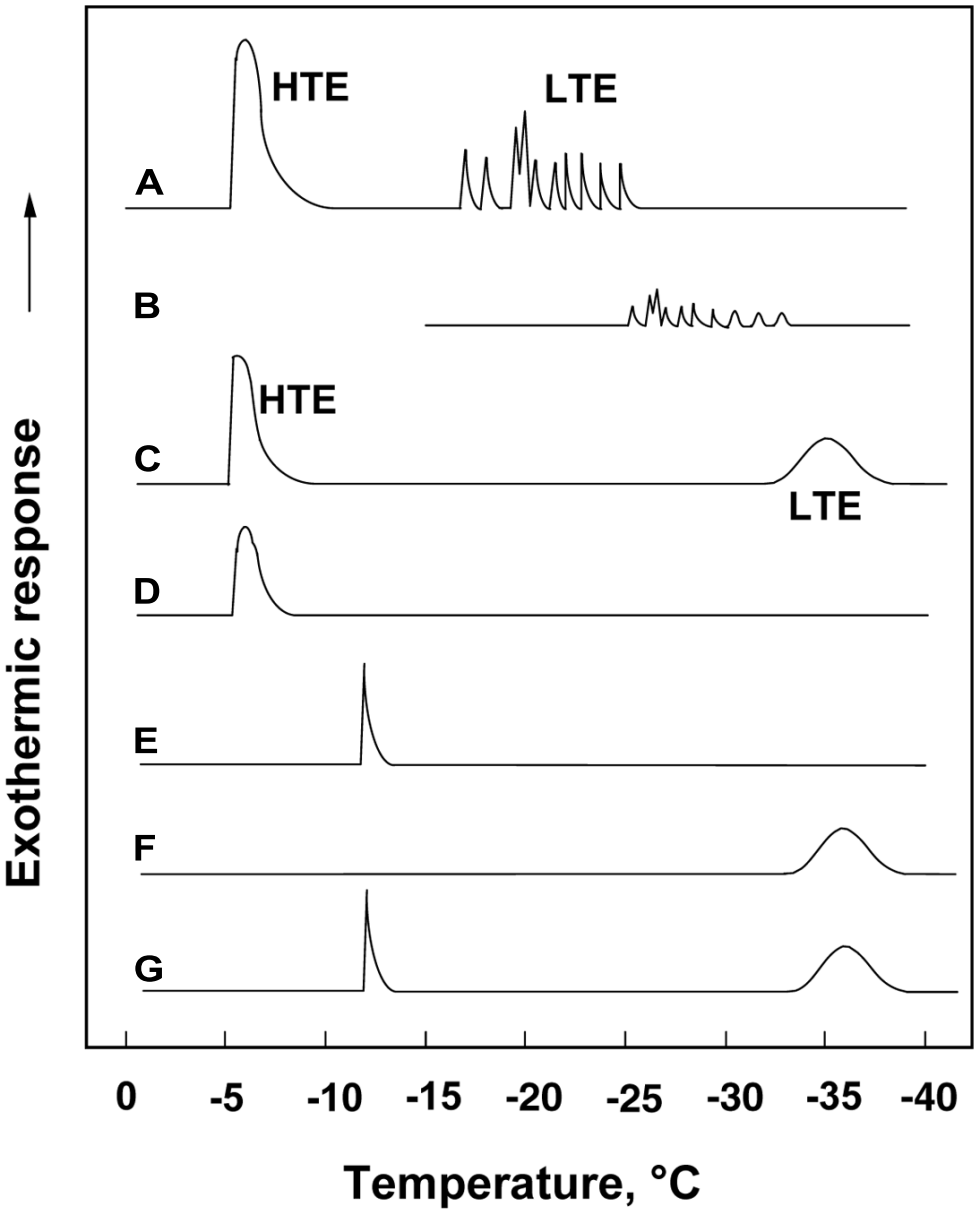
**(A)** A typical DTA profile of an *Rhododendron japonicum* flower bud with 1 cm twig (collected in early December) cooled at 3°C/h, showing a HTE at -5°C and spike-like LTEs between -17 and -24°C, which got smaller and shifted to lower temperatures when the flower bud was stored at -15°C for 3 days before being used for DTA without thawing **(B)**. A typical DTA profile of an intact current year 2.5 cm stem **(C)**, excised bark **(D)**, excised xylem showing a single exotherm peak at -12°C **(E)**, excised pith showing a broad exotherm starting around -33°C **(F)** and excised xylem with pith **(G)**.

**FIGURE 2:**
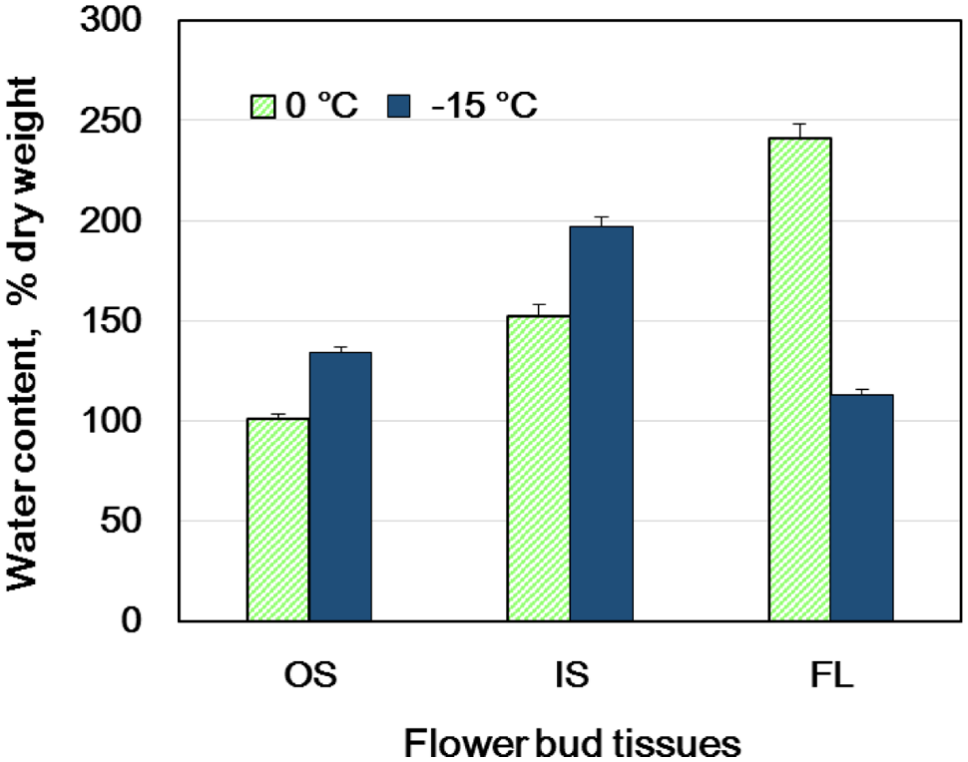
**Changes in the water content of flower bud tissues of wintering *R. japonicum* (collected in early December) during slow cooling (5°C/day) to -15°C and subsequent storage at -15°C for 3 days.** OS, outer scales; IS, inner scales; FL, florets. The water content is expressed on a dry weight basis (mean ± SE, *n* = 4).

**FIGURE 3:**
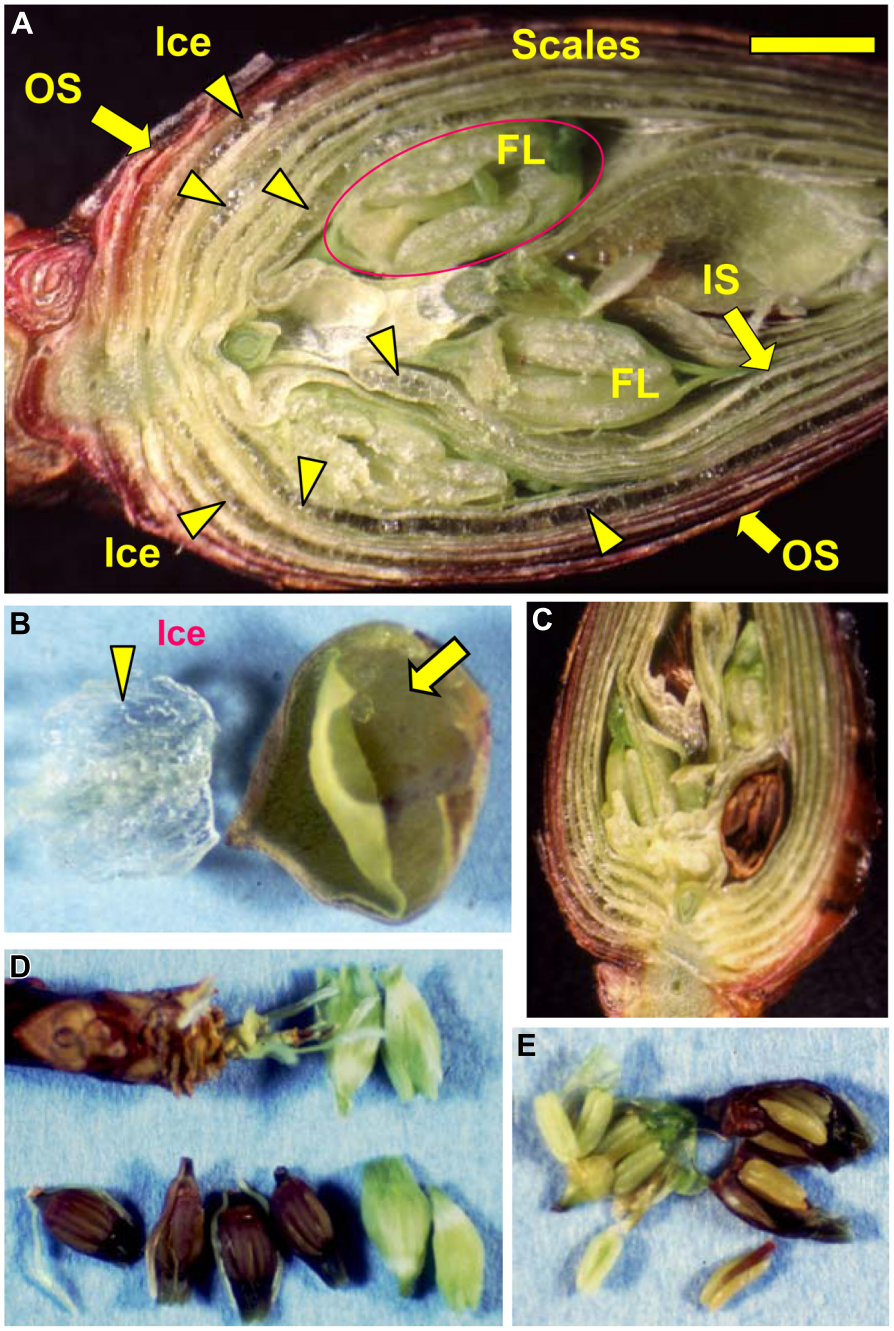
**Microscopic observation of a flower bud of *R. japonicum* (collected in early December) stored at -15°C for 3 days following slowing cooling **(A)**.** Icicles located within the outer or inner scales are denoted by the arrow heads. OS, outer scale; IS, inner scale; FL, floret. The bar = 2 mm. **(B)** Accumulated ice was removed from an outer flower bud scale with forceps, a large bag-like cavity that had been formed by the ice formation within the scale was then clearly evident (arrowed). Freezing of supercooled florets (releasing LTE as shown in ****Figure 1A****) resulted in all or nothing type of injury in the florets which turned brown **(C,D)** with all the tissues in the floret injured **(E)** whilst the unfrozen florets remained viable and green **(C–E)**.

Previously we have shown that wintering flower buds of *R. japonicum* undergo typical extraorgan freezing in response to slow cooling to subfreezing temperatures using materials cultivated in Sapporo (Ishikawa and Sakai, 1981). To check if the extraorgan freezing is affected by the collection sites, we conducted DTA (**Figure 1**), water content determination (**Figure 2**) and microscopic observation of buds slowly cooled to -15°C (**Figure 3**) using wintering flower buds randomly collected from the natural populations in a mountain area (1100 m elevation) of Tochigi prefecture (middle part of Japan). These wild* R. japonicum* plants have more diversity in the morphology of flower bud scales (number and size) than the cultivated variety and are suitable for checking the consistency in the ability to undergo extraorgan freezing.

When flower buds were cooled at 3°C/h, they produced a large HTE around -5°C (likely representing the freezing of bud scales and stem bark) and about ten LTEs around –17 ∼-24°C (**Figure 1A**), each peak of which corresponded well with all or nothing type injuries of individual florets (**Figures 3C–E**). When the flower buds were cooled at 5°C/day to -15°C and stored for 3 days at -15°C, the cold hardiness of the florets was rapidly increased, which corresponded well with the shift of the LTE range from ∼-24 to ∼-32°C (**Figures 1A,B**). During this process, the water content of the florets rapidly decreased whilst those of outer and inner bud scales increased (**Figure 2**). Correspondingly, the buds exposed to this condition had icicles accumulated within the outer scales and inner scales (**Figure 3A**) whilst the florets remained unfrozen but got dehydrated. Ice accumulation created large cavities within the outer and inner bud scales especially in the basal half of the scales (**Figure 3B**). This resulted in the bag-like appearance of the bud scales, which only emerged after experiencing subfreezing temperatures and the cavities got larger by repeated freezing. Such icicle accumulation in the bud scales of many plant species was first documented by Wiegand (1906) and Dorsey (1934), and later by Quamme (1978).

These results are consistent among flower buds with diverse bud scale morphology in the natural populations (data not shown) and also with the previous observation of extraorgan freezing in flower buds sampled from *R. japonicum* plants cultivated in Sapporo (Ishikawa and Sakai, 1981). The results are also in agreement with the results of NMR micro-imaging (Price et al., 1997), where the spontaneous freezing of bud scales, bark and xylem was observed to occur by -7°C, and dehydration of the florets proceeding through the pedicel (the basal part of florets). Flower buds collected from other sites also displayed similar extraorgan freezing (data not shown). The plants used from each collection site have been grown there for more than 10 years and likely fully adapted to the climatic conditions of each site. Thus, the employment of extraorgan freezing as the freezing behavior is likely the property of the species and the effect of collection sites or their environments seems minimal. Alternatively, the extent of water migration was more pronounced in the buds in early winter and early spring when the floret water content was high (data not shown), which was consistent with our previous studies (Ishikawa and Sakai, 1981, 1985). With the mechanism of extraorgan freezing (more extensive water migration from the florets to the scales than other *Rhododendron* species), *R. japonicum* can overwinter with only marginal injury to the florets in the eastern parts of Hokkaido where the air temperature frequently drops to -30°C (Ishikawa and Sakai, 1981).

### Spatial Distribution of INA within the Flower Buds and Effect of Collection Sites

An unsolved question in the mechanism of extraorgan freezing in flower buds is why bud scales freeze first before florets despite their much lower water content (**Figure 2**). To answer this question, we determined the INA of flower bud tissues in late autumn or early winter using the revised test tube method (Kishimoto et al., 2014a,b). In the test tube INA assay, the detached flower bud parts are exposed to a homogeneous temperature in the circulating bath and the initiation of freezing in the tissue sample was detected as the freezing of all of the water (2 mL) in a tube. Consequently, the ability of each tissue to cause heterogeneous ice nucleation can be fairly compared (Kishimoto et al., 2014b). This is not necessarily realized in DTA or thermography analyses of intact organs, where the outer tissues are often exposed to external ice or lower temperatures than the inner tissues. The test tube method also allows both quantitative and qualitative analyses of the INA by changing the amount of each sample and its exposure to different treatments. It may also allow the discrimination of tissue freezing due to their own INA from freezing initiated from ice propagation from nearby tissues with high INA, which otherwise is not readily distinguishable in DTA.

**FIGURE 4:**
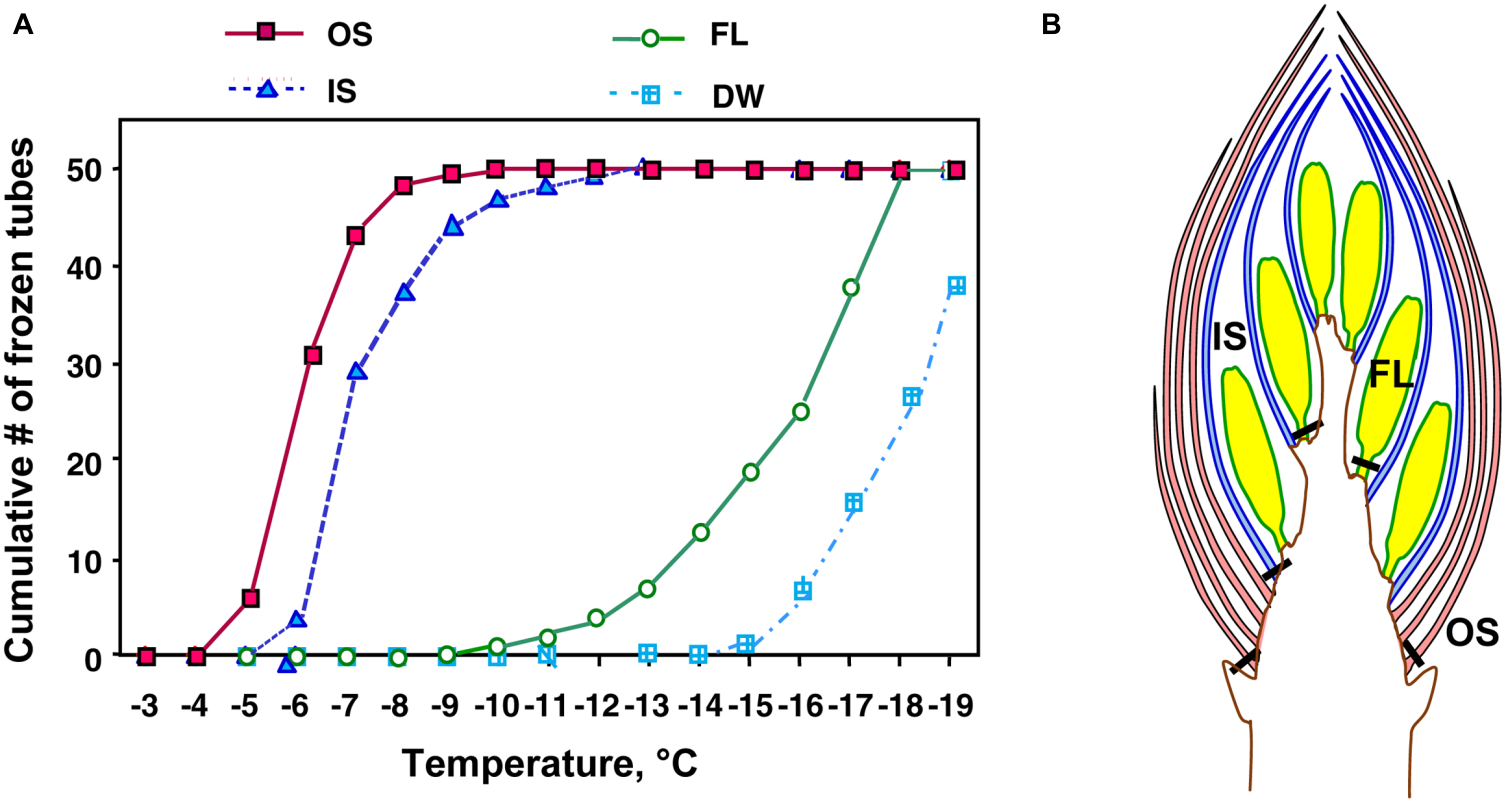
**A typical result of test tube-based INA assay **(A)** of outer scales (OS), inner scales (IS) and florets (FL) excised at the positions shown in **(B)** from wintering *R. japonicum* flower buds collected in early December.** Tubes containing a single outer scale, inner scale or floret of *R. japonicum* in 2 mL autoclaved Milli-Q water were cooled in 1°C decrements (1°C in 25 min) to -20°C. DW, autoclaved Milli-Q water without the specimen.

Among the tissues of *R. japonicum* flower buds, the outer bud scales and the inner scales showed high levels of INA (median INT) with narrow variance of INT whilst the florets had low levels of INA with a larger variance of INT (**Figure 4**). The median INT of the outer scales (-5 ∼-6°C) corresponded well with the initiation temperature of HTE (**Figure 1A**). The inner scales are coupled with the florets and have approximately 50 ∼ 70% of the dry weight of outer scales with a greater variance in mass than the outer scales. This likely resulted in the greater SD and slightly lower median INT in the inner scales (**Table 1**; **Figure 4**). When four times the amount of inner scales were used per test tube in the INA assay, the median INT became closer to that of a single outer scale (**Table 2**).

The INT of florets was low but not in agreement with the LTE initiation temperatures (**Figures 1A,B**). Since the control tubes (i.e., without samples) in this INA assay system froze below -15°C (**Figure 4**), the INA levels lower than this range may not have been properly measured. The freezing temperature of the control tubes can be lowered by reducing the volume of water in the assay system: tubes with 20 μL of water remain unfrozen at -20°C. Yet, smaller water volumes restrict the size of tissue samples that can be added. An alternative method is to use homogenized sample suspension in droplets (Brush et al., 1994), however, this may destroy the INA responsible substances or structures and also allow the leakage of cellular contents that likely have anti-nucleating activity (ANA: the ability to suppress INA), which will be detailed later. The smaller volume assay is prone to be more affected by the presence of ANA than the larger volume assay (for the same amount of tissue) since the concentration of ANA substances released from the tissues into the assay water becomes higher. The factors determining the low INT ranges of plant tissues remain to be further investigated.

Similar distinct differences in the INA levels and variance between bud scales and florets were observed regardless of collection/cultivation sites or whether they are cultivated varieties or from natural populations with more diverse bud morphologies (**Table 1**). The INA distribution within the flower buds clearly demonstrates that bud scales have the ability to freeze before the florets in spite of the lower water content in the bud scales (**Figures 1A,B, 2**, and **4**). The outer most localization of bud scales within a bud may help freeze first but the results confirm that they have the intrinsic capability to ice-nucleate at warmer subzero temperatures, acting like a subfreezing temperature sensor (Kishimoto et al., 2014a,b). In contrast, the low levels of INA in florets likely helps the florets remain unfrozen and stably deep-supercooled. Once the bud scales initiate freezing, there is a reduction of chemical potential in the bud scales, which likely allows the bud scales to withdraw water from the unfrozen florets and accumulate icicles within the scales (**Figures 3A,B**). The INA distribution within the flower buds (high in bud scales and low in florets) likely plays an important role in the initiation and subsequent process of extraorgan freezing (Ishikawa and Sakai, 1985).

### Effect of Sample Amount, Grinding and Autoclaving on Bud Scale INA Levels

To characterize the INA in bud scale tissues, the amount of bud scales in the INA assay tubes was increased by fourfold (**Table 2**). The INA levels (median INT) of the outer and inner bud scales increased by 0.4 and 0.7°C, respectively. The variance of INT became narrower in the inner scales. When the outer scales were homogenized into powder using a mortar and pestle, the ground powder added to the assay tubes at the same ratios (equivalent to a single or four bud scales) retained the similar levels of INA (**Table 2**).

To check the effect of autoclaving on the INA of bud scale tissues, the frozen assay tubes with samples inside (used in the first INA assay) were thawed at room temperature and autoclaved at 121°C for 15 min before being used for the second INA assay. Autoclaving decreased the median INT of a single outer and inner bud scale by 0.7 ∼ 1.6 and 1.0 ∼ 2.1°C, respectively (**Tables 1** and **2**). Following the second INA assay, the tubes were allowed to thaw and the supernatant of the autoclaved samples was aseptically replaced with fresh sterilized water (2 mL) prior to the third INA assay. Interestingly, the water replacement resulted in increases in the median INT of outer and inner bud scales by 0 ∼ 1.0 and 0.5 ∼ 1.7°C, respectively, compared to the second INA assay results (autoclaved samples; **Tables 1** and **2**). This eventually allows the bud scale samples to recover INA levels similar to those of intact samples, but more precisely, the extent of recovery varied depending on the season and the tissues as detailed later.

The results of grinding and autoclaving treatments imply that the INA in the bud scales is not derived from the macro structures such as trichomes and is likely attributed to some substances that are resistant to autoclaving (121°C for 15 min). Comparison of the first (intact specimen) and third (i.e., autoclaved and water-replaced samples) INA assays clearly discriminates the levels of sensitivity or resistance of the INA to autoclaving.

### Occurrence of Anti-Nucleation Activity (ANA) in the Supernatant of Autoclaved Samples

The autoclaving-induced INA decreases and the subsequent INA recovery by water replacement observed in the bud scales can be interpreted as follows. The supernatant (leachate from the autoclaved bud scales) likely has ANA, defined as the ability to suppress the INA of a substance or tissues (Ishikawa et al., 2009), which can be removed by the replacement of supernatant with sterilized water. To roughly estimate the concentration of solutes in the supernatant of the bud scales, the osmolality was determined. Osmolality was less than10 mOsm/kg. This implies that the detected ANA is different from the colligative effects of the solutes (freezing point depression) and the mechanism remains to be investigated biochemically and biophysically. Similar ANA has been observed in the autoclaving-derived leachate from about 70% of the plant tissues of over 600 species that we surveyed for the occurrence of INA in plants ranging from tropical to boreal regions (Ishikawa, 2014). The ANA was widely observed in tissues, irrespective of their origins, woody or herbaceous and levels of cold hardiness. Even the leachate from tropical plant tissues showed high ANA levels and this is likely a general phenomenon (Ishikawa, 2014). Yet, high ANA levels have been reported in the leachate from the leaves of a cold hardy palm, *Trachycarpus fortunei*, which employs deep-supercooling as the cold hardiness mechanism (Ishikawa et al., 2009).

### Seasonal Changes in the INA of Flower Bud Tissues

**FIGURE 5:**
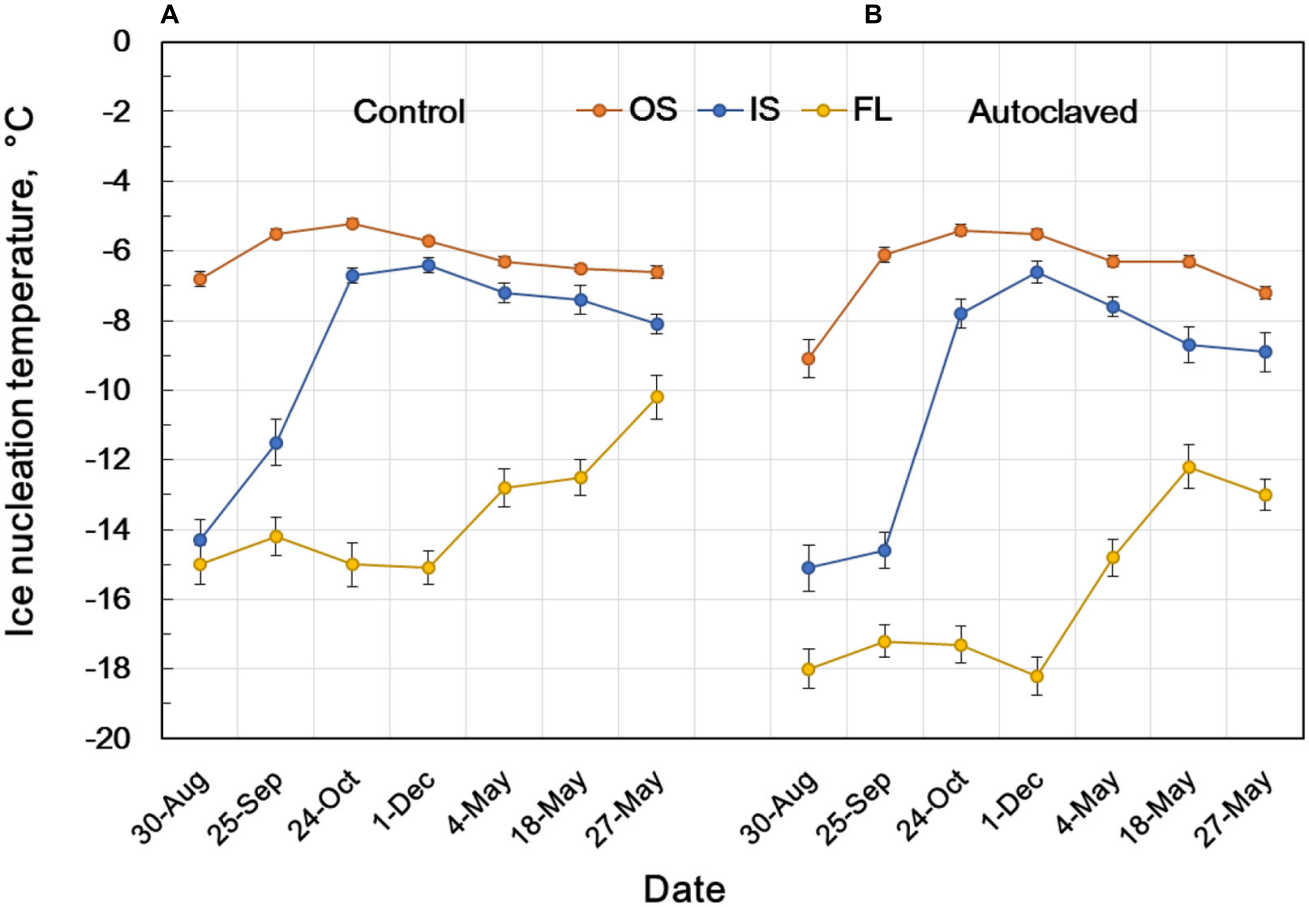
**Seasonal changes in the INA of intact flower bud parts of *R. japonicum***(A)** and INA after autoclaving and replacement of water **(B)** showing the stability of INA at 121°C for 15 min.** INA was determined as the 50% ice nucleation temperature (median INT) using the test tube INA assay as shown in ****Figure 4****. OS, outer scale; IS, inner scale; FL, floret. The data are mean ± SE (*n* = 3).

To know how the INA in flower bud scales develops and seasonally changes, the INA in flower bud tissues were followed from August until May except for mid-winter (the plants were covered with snow from January until early April) using the natural populations in Tochigi Prefecture (**Figure 5**). Flower bud morphogenesis starts from late June or early July and comes to an end in late August or early September. The INA level of the outer bud scales in late August was about -7°C and increased to the highest level (-5.2°C) in late October when they experience the first autumnal frost or freeze. In contrast, the INA level of the inner bud scales was low (> -14°C) in late August, but rapidly increased to -6.7°C in late October and to -6.4°C in early December (**Table 1**; **Figure 5**), just in time for their frequent exposure to subfreezing temperatures when a freezing sensor function is required. The INA in the outer and inner bud scales remained at high levels during the winter but it gradually declined in the spring (May 4 ∼ May 27) when flower buds resumed growth (the bud scales still attached). The INA in the floret stayed at low levels from August until the end of winter but it went up in the spring as the floret dry weight rapidly increased by 1.5- (May 4), 3- (May 18), and 6-fold (May 27).

Comparison of the INA levels between intact samples (first INA assay) and the autoclaved/water-replaced samples (third INA assay) indicates the seasonal alterations in the autoclaving resistance of the bud scale INA (**Figure 5**). The INA in the outer scales was moderately sensitive to autoclaving (2°C decrease) in late August but became resistant to autoclaving in late September until the middle of May. On the other hand, the INA in the inner scales was sensitive to autoclaving from late August until late October (1 ∼ 3°C decrease) but became resistant from early December until early May. These tendencies in the seasonal changes and the autoclave sensitivity of INA were consistent in the 10 year consecutive measurements of flower bud INA in the natural populations in Tochigi.

Similarly, blueberry stem bark tissues achieve the maximal INA level just before the first autumnal frosts or freeze (October ∼ middle November) although their maximum INA level is much higher (-1 ∼-2°C; Kishimoto et al., 2014a,b). Yet, the INA levels in blueberry stems greatly decrease as they experience repeated freezing in winter and go up in March (Kishimoto et al., 2014a).

### Freezing Behavior of *R. japonicum* Stem Tissues

When wintering current year stem segments were cooled at 3°C/h, they produced an HTE (starting at around -5°C) and a broad LTE (starting around -33°C; **Figure 1C**). Excised xylem plus pith (bark tissues completely removed) had a sharp HTE at -12°C and a broad LTE at the same temperature range as the intact stem (**Figure 1G**). Excised pith (xylem removed) had no HTE and an LTE only (**Figure 1F**) whilst excised xylem tissues had only a sharp peak around -12°C (**Figure 1E**). Excised bark tissues had an HTE around -5°C (**Figure 1D**). These results indicate that the HTE of intact stems likely represents the freezing of bark and xylem whilst the LTE represents the freezing of deep-supercooled pith.

It is possible that the xylem ray parenchyma of *R. japonicum* do not undergo deep supercooling (**Figure 1E**) unlike other typical temperate woody species (Quamme et al., 1972; George and Burke, 1977; Wisniewski, 1995). Supportively, MRI studies of* R. japonicum* (Price et al., 1997) revealed that the image signal intensity in the xylem reduced to low levels at -7°C whilst the intensity in the pith persisted at the same level till -21°C as the unfrozen control (4°C). This was in contrast to the xylem retaining high levels of signal intensity in MRI images at -21°C in peach (unpublished result) and maple (Ishikawa et al., 1997). The pith tissues of wintering *R. japonicum* are alive (green-colored) with thick cell walls and starch accumulating plastids. The pith of this species is a good material to study the mechanism of deep supercooling since the whole pith tissues remain unfrozen to as low as -33°C, which is their intrinsic freezing behavior. In xylem tissues of other typical temperate woody species, only ray parenchyma (scattered in the xylem) remain supercooled and the majority of neighboring xylem vessels are frozen (Hong and Sucoff, 1980). This often makes it difficult to study the mechanism of deep supercooling or to conduct pin-point biochemical and molecular analyses of the tissues that deep-supercool.

### Spatial Distribution of INA in the Current Year Stem Tissues

The bottom four rows of **Table 1** summarize the INA of the stem tissues in early winter. Within the stem, bark tissues had high INA (-6°C), which approximately coincided with the HTE initiation temperature (**Figures 1C,D**) whilst the xylem and pith had much lower INA (-12 ∼-13°C). The high INA in the bark corresponds well with the intrinsic freezing behavior (extracellular freezing) of the bark as visualized in MRI studies (Price et al., 1997). The INA of the xylem coincided with the HTE initiation temperature of the excised xylem (**Figure 1E**). The low INA in the xylem likely explains why the xylem with ample free water in their vessels does not freeze first in the excised xylem. The freezing of xylem in wintering current year stems likely originates from the bark tissues since the INA in the bark is much higher than the xylem, which resembles the case of blueberry stems (Kishimoto et al., 2014a,b).

Since the pith cells do not undergo extracellular freezing but remain deeply supercooled, they do not require high levels of INA outside the cells whilst low levels of INA are favored inside the cells. In this sense, the lack of efficient INA in the pith was in agreement with their intrinsic freezing behavior (deep supercooling). Yet the INA levels (-12 ∼-13°C) was much higher than the initiation temperature (∼ -33°C) of pith tissues (**Figure 1F**). This could be due to contamination of xylem residues or from other origins arising from the tissue excision, or due to the intrinsic problem of the assay system as discussed earlier. This remains to be further investigated.

### Effect of Autoclaving on the INA of Stem Bark and the Occurrence of Two Distinct Types of INA Within the Plant

The responses of stem tissues to autoclaving/water replacement are also shown in the last four rows of **Table 1**. Autoclaving (121°C for 15 min) drastically decreased the INA in the stem bark by 7°C (-6.2 →-13.3°C) whilst the INA levels of xylem and pith were low in the intact stem and affected only slightly (0.1 ∼ 1.5°C) by autoclaving. The results show that the INA levels of the bud scales and stem bark are in a similar range (-5 ∼ -6°C) but differ considerably in the sensitivity to autoclaving. Two distinct types of INA exist within the *R. japonicum* plant: autoclaving-resistant type in the bud scales and autoclaving-labile type in the bark. Whether these two types are related or should be attributed to completely different compounds remains to be investigated. The seasonal increases in the autoclaving resistance of the INA in the bud scales from summer to winter imply that the autoclaving-sensitive INA may be converted to resistant type (**Figure 5**). The INA in blueberry bark tissues and *Prunus* stems has also been known to be sensitive to autoclaving or heat treatment (Gross et al., 1988; Kishimoto et al., 2014b).

### Seasonal Changes in the Osmolality of Flower Bud Tissues

**FIGURE 6:**
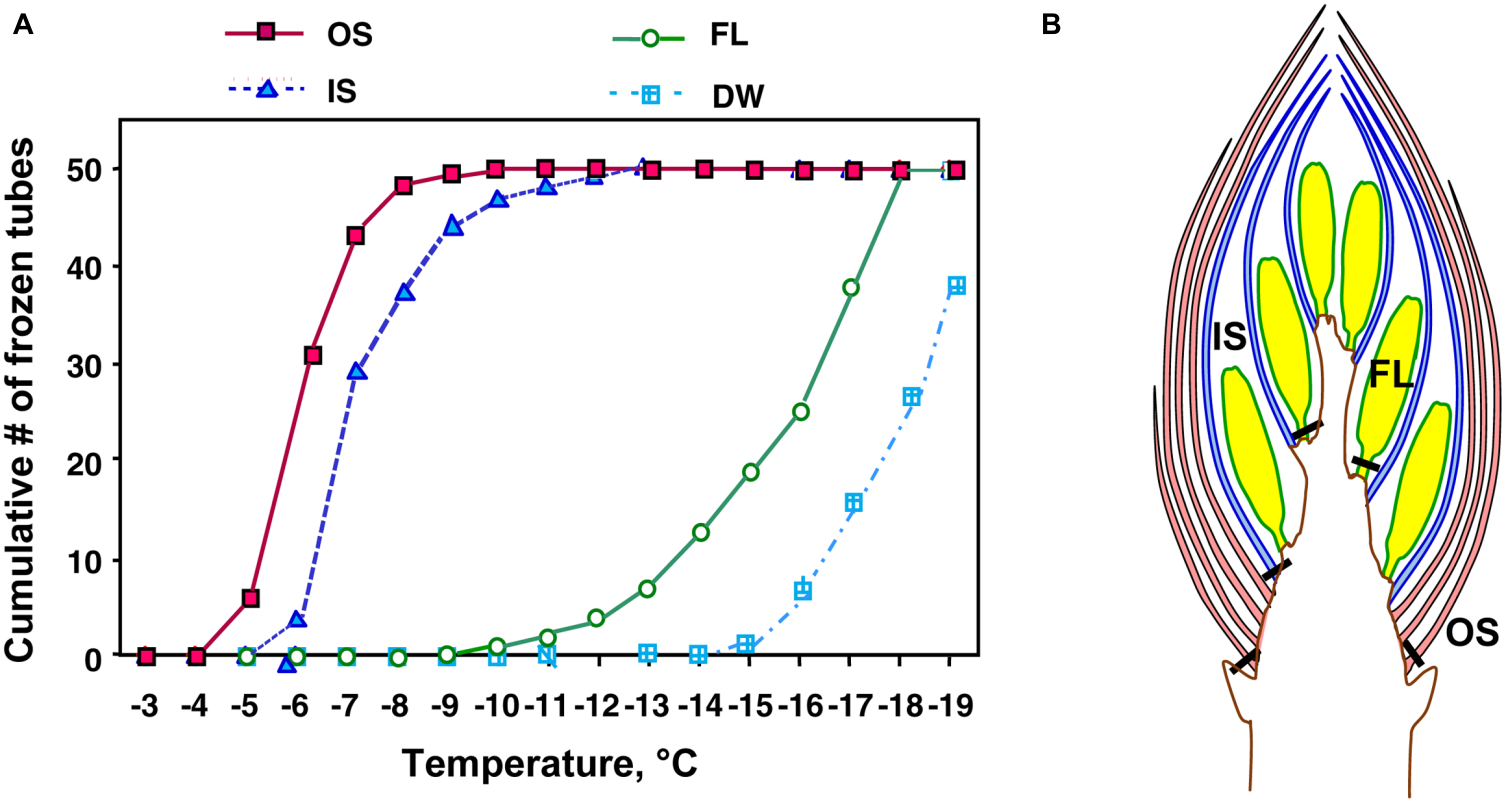
**Seasonal changes in the osmolality of flower bud tissues of *R. japonicum*.** OS, outer scale; IS, inner scale; FL, floret. The data are mean ± SE (*n* = 4). The error bars that are smaller than the symbols are not indicated.

To explore some other factors that may contribute to the mechanism of extraorgan freezing, temporal alterations in the osmolality of flower bud scales and florets were determined at monthly intervals (**Figure 6**). In early September when the flower bud formation came to an end, the bud scales and florets had similar osmolality levels (approximately 400 mOsm/kg), yet in the following order: florets > inner bud scales > outer bud scales. In late September, the osmolality of the outer and inner scales remained at similar levels whilst that of florets was almost doubled (800 mOsm/kg) without experiencing frosts. The osmolality of the bud scales steadily increased to 800 mOsm/kg by early December, during which the plants in the collection site were exposed to autumnal frosts starting from the middle of October. In the meantime, the osmolality of the florets reached 1200 mOsm/kg by early November and remained at that level until early December. This approximately agrees with the freezing point depression of -1.8°C (equivalent to about 1000 mOsm/kg) reported for the homogenate of florets collected in December in Sapporo (Ishikawa and Sakai, 1981). The results clearly demonstrate that the floret osmolality was kept about 400 mOsmol higher than in the outer and inner bud scales (florets >> inner bud scales > outer bud scales) from late September until early December (**Figure 6**). The 400 mOsm/kg differences are equivalent to the florets having about 0.7°C lower freezing point than the bud scales. This could be another factor contributing to the mechanisms of extraorgan freezing. The higher osmolality in florets may help them remain unfrozen and slow the rate of water migration from the florets to the scales due to the reduced differences in the chemical potential between the frozen scales and the florets with increased osmolality.

On the other hand, the estimated freezing point difference (about 0.7°C or less) does not explain the contrasting differences (bud scales vs. florets) in the INA levels (**Table 1**; **Figure 4**) and the freezing behaviors (HTE vs. LTE; **Figure 1A**). The seasonal increases in the osmolality (freezing point depression) do not correlate with the seasonal INA changes in the bud scales and florets (**Figure 5** vs. **Figure 6**). How the osmolality difference interacts with the INA distribution remains to be investigated as the osmolality represents the intracellular osmoticum whilst the INA represents the INA in the extracellular space. The results in **Figures 5** and 6 clearly depict that freezing behaviors in wintering *R. japonicum* flower bud tissues are primarily governed by the INA levels of the tissues rather than their osmolality.

### Detection of Microorganisms Associated with Bud Scales of Wintering Flower Buds

The INA of microbial origin, such as ice nucleating bacteria and fungi, is well-known (Ashworth and Kieft, 1995; Hirano and Upper, 1995). To check the possibility of the INA within the flower bud scales being of microbial origin, we attempted to detect microorganisms associated with flower bud scale tissues of *R. japonicum* collected in late October when the highest levels of INA were achieved in outer and inner bud scales (**Figure 5**). We vigorously washed the whole flower buds to remove loosely associated microorganisms on the surface of buds. Microbial colonies were detected in 14 outer bud scales out of 15 samples incubated on half strength cornmeal agar medium at 25°C for 2 weeks. In contrast, out of 15 inner bud scales incubated on the medium, microbial colonies were detected with only two inner scales (**Table 3**). The result does not exclude the possibility of inner scales still associating microorganisms that cannot grow on the medium. Yet, it implies that the inner scales are relatively clean compared to the outer scales and that the INA within the inner scales is unlikely of microbial origin.

**Table 3:** Detection of microorganisms (mainly fungi) associated with the flower bud scales of *R. japonicum*.

*R. japonicum* Tochigi October 24	The number of fungi + petri dish	
	OS	IS
FB1	3/3	1/3
FB2	3/3	0/3
FB3	2/3	0/3
FB4	3/3	0/3
FB5	3/3	1/3
**Total**	14/15	2/15

OS, outer scales; IS, inner scales; FB, flower buds. For the details, refer to the **Materials and Methods** section.

## General Discussion and Conclusion

The present study was undertaken to address the mechanisms involved in extraorgan freezing in flower buds by determining INA and osmolality of the tissues: why bud scale tissues freeze before the florets and why the florets remain unfrozen through stable deep supercooling. The results support the hypothesis that the bud scales have higher INA levels than the florets, irrespective of the collection sites (Ishikawa and Sakai, 1985). This ensures that the bud scales initiate freezing first at warmer subzero temperatures, resulting in the reduction of chemical potential in the bud scales, which likely allows the migration of floret water to the bud scales and the accumulation of icicles within the bud scales. In contrast, the low levels of INA in florets likely helps the florets remain unfrozen through stable deep supercooling. The higher osmolality in the florets may also help them remain unfrozen and it may also contribute to retarding the water migration from the florets to the scales.

However, to establish extraorgan freezing, there must be a barrier that prevents ice propagation from the bud scales into florets but is permeable to water (water or vapor) from the florets to the bud scales (Ishikawa and Sakai, 1981, 1985). Such a barrier has been postulated to be located in some place between the basal part of florets and the basal part of bud scales (Ishikawa and Sakai, 1981; Price et al., 1997). Precise localization and determination of the identity of the barrier (biochemical, structural in nature or just a dehydrated zone) remain to be elucidated (Quamme, 1978; Ishikawa and Sakai, 1981, 1985; Ashworth, 1992; Chalker-Scott, 1992; Kader and Proebsting, 1992; Wisniewski et al., 2009; Kuprian et al., 2014).

The fine distribution of INA within *R. japonicum* stems corresponds well with the intrinsic freezing behaviors of the stem tissues: the high INA in the bark is linked to the extracellular freezing of the tissues whilst the low INA in the pith agrees with the deep supercooling of the pith. The widely held view (Sakai and Larcher, 1987) that the xylem is the primary site for ice nucleation is likely downplayed by the finding of low INA in the xylem. Instead, the freezing of xylem likely originates from the bark (Kishimoto et al., 2014a,b). The results also depict that the tissues can be classified into three types in terms of INA: the tissues (bud scales and bark) with high INA levels that freeze autonomically at warmer subzero temperatures, the tissues (xylem) with lower INA levels whose freezing originates from the other spontaneously frozen tissues and finally the tissues (florets and pith) with low INA levels that remain unfrozen. Together, the order of freezing in wintering plant tissues (from the primary freezing tissue to the last tissue remaining unfrozen) seem to be closely correlated with the order of INA levels in the tissues rather than their osmolality. The high INA in the flower bud scales and stem bark likely function as the subfreezing sensor that ensures initiation of freezing of these tissues at warm subzero temperatures (Kishimoto et al., 2014a,b) so that the intrinsic freezing behaviors (extraorgan freezing or extracellular freezing) can be achieved. And yet, questions such as which freezes first, bud scales or stem bark (the bud scales being likely the first as indicated by the slightly higher INA levels) and how the freezing initiated in the bud scales spreads to the stem or vice versa in natural conditions remain to be solved by visualizing the freezing process using infrared thermography.

The results of autoclaving experiments (121°C for 15 min) indicate that two distinct types of INA exist within the *R. japonicum* plant: autoclaving-resistant type in the bud scales and autoclaving-labile type in the bark. These two types could be related as implicated by the temporal increases in the autoclaving resistance of the bud scale INA. The INA in the bud scales is not derived from the macro structures such as trichomes as it is unaffected by tissue grinding and is likely attributed to some substances that are resistant to autoclaving (121°C for 15 min). This is in contrast to the heat labile property (inactivated at 80°C) of the known microbial INA (Ashworth and Kieft, 1995; Hirano and Upper, 1995). These properties clearly indicate that the bud scale INA of wintering *R. japonicum* flower buds is likely of intrinsic origin rather than microbial origin. Autoclaving/water replacement experiments implicated the occurrence of ANA in the leachate of autoclaved bud scales, which suppress the INA at concentrations less than 10 mOsm/kg. The ANA is obviously different from the colligative effects of the solutes and widely observed in tissues, irrespective of their origins, woody or herbaceous and levels of cold hardiness (Ishikawa, 2014).

Seasonal changes in the INA in flower bud tissues reveal that the INA in bud scales increased from late August to late October just in time for the first autumnal frosts when a freezing sensor function is required to survive the first freeze. It appears that some substances responsible for the INA are actively synthesized in the bud scales. It is known that the first autumnal frosts accelerate cold acclimation (Kuroda et al., 1990) and surviving the first frosts is likely important. Freezing is a physical event but the INA in bud scales is a biological trait that contributes to the primary freeze in the bud scales. As shown in this study, the test tube INA assay is useful for determining the fine spatial and temporal localization of INA in plant organs, which greatly helps clarify the mechanisms governing diverse freezing behaviors intrinsic to the tissues. The results also indicated an intrinsic problem in the INA assay system used in this study: the INA levels lower than -15°C (freezing range of the control tubes without samples) may not be properly evaluated.

## Conflict of Interest Statement

The authors declare that the research was conducted in the absence of any commercial or financial relationships that could be construed as a potential conflict of interest.

## Abbreviations

ANA: anti-nucleation activity
DTA: differential thermal analysis
FL: floret
HTE: high temperature exotherm
INA: ice nucleation activity
INT: ice nucleation temperature
IS: inner scale
LTE: low temperature exotherm
OS: outer scales.
